# Limb salvage in diabetic foot necrotizing fasciitis complicated by extensively drug-resistant *Acinetobacter baumannii*: a case report of an integrative treatment approach

**DOI:** 10.3389/fendo.2026.1936922

**Published:** 2026-08-26

**Authors:** Yecen Tian, Yue Shi, Peng Liu, Qiang Xu, Changhai Zhang, Yunsha Zhang

**Affiliations:** 1 School of Integrative Medicine, Tianjin University of Traditional Chinese Medicine, Tianjin, China; 2 Department of Surgery, Binhai New Area Hospital of Traditional Chinese Medicine, Tianjin, China; 3 Traditional Chinese Medicine (TCM) Institute of Sore and Ulcer, Tianjin University of Traditional Chinese Medicine, Tianjin, China; 4 Tianjin Institute of Traditional Chinese Medicine Surgery, Tianjin, China; 5 Department of Traditional Chinese Medicine Surgery, The Second Affiliated Hospital of Tianjin University of Traditional Chinese Medicine, Tianjin, China

**Keywords:** diabetic foot ulcer, extensively drug-resistant *Acinetobacter baumannii*, integrative medicine, limb salvage, necrotizing fasciitis

## Abstract

**Background:**

When diabetic foot ulcers (DFUs) complicated by necrotizing fasciitis (NF) show persistent culture positivity for extensively drug-resistant *Acinetobacter baumannii* (XDR-AB), systemic antimicrobial options are severely limited, posing a severe challenge to limb salvage. Exploring integrative intervention strategies in this setting is of great clinical significance.

**Case presentation:**

A 59-year-old patient was admitted with a 20-day history of a right foot ulcer and a 10-year history of poorly controlled type 2 diabetes mellitus. The patient was initially diagnosed with diabetic foot ulcer, which progressed to lower-limb necrotizing fasciitis and septic shock requiring intensive care unit (ICU) supportive treatment. Due to persistent recovery of XDR-AB from wound cultures and severely limited effective systemic antibiotic options, the team used traditional Chinese medicine (TCM) as adjunctive therapy alongside standard debridement, vacuum sealing drainage (VSD), and intensive care support. Following the intervention, the patient’s systemic inflammatory markers gradually stabilized despite persistent culture positivity, without recurrent sepsis. With the progressive clearance of necrotic tissue and the growth of healthy granulation tissue, the team successfully performed staged skin grafting while wound cultures remained positive on days 65 and 109, with complete graft take after both procedures. After 115 days of comprehensive treatment, the right lower limb wound stabilized, and the limb was successfully salvaged, allowing for a smooth discharge.

**Conclusion:**

For complex cases of DFUs complicated by NF and XDR-AB culture positivity where antibiotics are severely restricted, the addition of TCM to standard multidisciplinary care was associated with infection control and wound repair and may represent an exploratory limb-salvage approach. However, its generalizability and exact efficacy require further validation through subsequent research.

## 1 Introduction

Diabetes mellitus is a metabolic disease characterized by chronic hyperglycemia, and its continuously rising prevalence has made it a major global public health issue (1) Diabetic foot ulcers (DFUs) are among the most severe complications of diabetes. Approximately 19% to 34% of individuals with diabetes will develop foot ulcers during their lifetime, of which 50% to 60% become infected. Among patients with moderate-to-severe infections, approximately 20% ultimately require amputation, and the 5-year mortality rate for infection-related DFUs can reach over 30%, escalating to more than 70% following major amputation (2, 3).

Necrotizing fasciitis (NF) is an acute, rapidly progressive infection involving the fascia and deep soft tissues, characterized by an insidious onset, rapid progression, and a high mortality rate (4). Due to impaired immune function, microcirculatory impairment, and neuropathy, patients with diabetes have a significantly increased risk of developing NF, and once it occurs, the disease course is often more severe. In the context of diabetic foot infections, NF frequently leads to extensive tissue necrosis and irreversible limb damage, making it one of the leading causes of major amputation. Previous studies have shown that diabetic foot complicated by NF significantly increases the risk of amputation, with the amputation rate rising to as high as 72.4% (5).

Diabetic foot infections are predominantly polymicrobial, with common pathogens including *Staphylococcus aureus* (including *methicillin-resistant strains*), *Pseudomonas aeruginosa, Enterobacteriaceae*, and *Acinetobacter baumannii.* With the widespread use of antibiotics, the proportion of drug-resistant bacteria has been increasing annually. A single-center study reported that the detection rate of *Acinetobacter baumannii* in patients with diabetic foot infections is approximately 14% to 15% (6). This bacterium is a typical hospital-acquired pathogen, and the emergence of its multidrug-resistant (MDR), extensive drug-resistant (XDR), and pandrug-resistant (PDR) strains have severely limited anti-infective treatment options. Furthermore, it is closely associated with difficulties in infection control, delayed wound healing, and an increased risk of amputation (7–11).

DFU complicated by NF constitutes a limb-threatening infection, and the persistent isolation of XDR-AB further increases the difficulty of infection control and limb-salvage treatment. The clinical management of such patients remains challenging, and published experience with limb-salvage treatment is limited. Here, we report a patient with DFU complicated by NF, extensive lower-extremity involvement, and persistent isolation of XDR-AB. Following comprehensive management involving aggressive surgical debridement, VSD, antimicrobial therapy, wound management, and integrative Chinese and Western medical interventions, functional limb salvage was ultimately achieved, providing a clinical reference for the management of similarly complex, limb-threatening infections.

## 2 Case presentation

A 59-year-old man with a 10-year history of type 2 diabetes mellitus and poor glycemic control (HbA1c, 14.2%) was admitted with a 20-day history of a right foot ulcer. Physical examination revealed wet gangrene of the right fourth toe, with marked swelling along the lateral aspect of the metatarsophalangeal joint, increased skin temperature, and a foul odor. Laboratory examinations indicated severe infection and metabolic derangements. Computed tomography of the foot showed gas within the right fourth metatarsal head and phalanges, accompanied by extensive soft-tissue swelling and gas formation. The preliminary diagnoses included diabetic foot associated with type 2 diabetes mellitus (Wagner grade 4; IWGDF/IDSA infection grade 4), diabetic ketosis, osteomyelitis of the right foot, anemia, and hypoproteinemia.

Immediately after admission, comprehensive treatment was initiated, including empirical antimicrobial therapy with ceftazidime, glycemic control, nutritional support, and correction of metabolic derangements. Meanwhile, based on the traditional Chinese medicine principle of “constraining the lesion and reducing swelling,” Jinhuang San Tincture, as recorded in *Waike Zhengzong*, was applied topically, together with stepwise debridement. Serial wound cultures successively yielded *Klebsiella pneumoniae*, *Streptococcus agalactiae*, *Proteus vulgaris*, *Streptococcus mitis*, and *Streptococcus anginosus*. Despite adjustment of the antimicrobial regimen, systemic infection remained poorly controlled. On hospital day 5, debridement of the right foot was performed. Based on the intraoperative findings, necrotizing fasciitis (NF) of the right foot was definitively diagnosed, and deep wound exudate was collected for bacterial culture. Given the progression of the disease, antimicrobial therapy was escalated to meropenem plus vancomycin.

**Figure 1:**
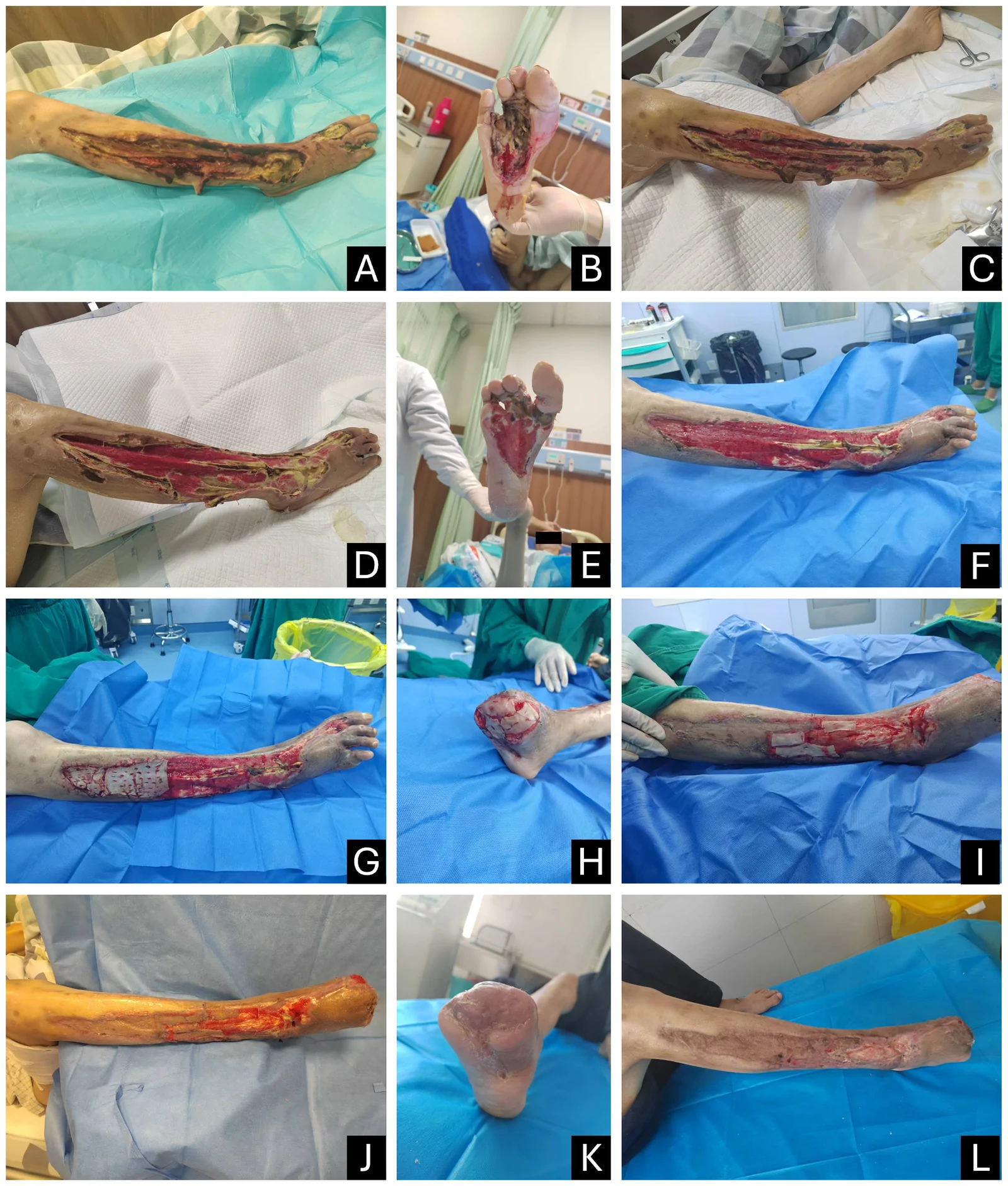
Case images. **(A, B, C)** Debridement of the lower extremity. **(D)** Treatment day 38. **(E, F)** First-stage skin grafting while wound cultures remained positive on day 65. **(G)** Post-first-stage skin grafting. (H, I) Post-second-stage skin grafting on day 109. **(J)** Survival of the second-stage graft by day 115. **(K, L)** Outpatient follow-up on day 155.

On day 7, the patient developed septic shock and was transferred to the intensive care unit (ICU). Despite aggressive critical care support, the local infection continued to progress, with swelling extending to the right lower extremity. The treatment team urgently performed extensive debridement and drainage of the lower extremity, and the intraoperative findings confirmed that NF involved the right lower extremity (**Figures 1A–C**). After clinical stabilization, the patient was transferred back to the general ward on day 11.

**Figure 2:**
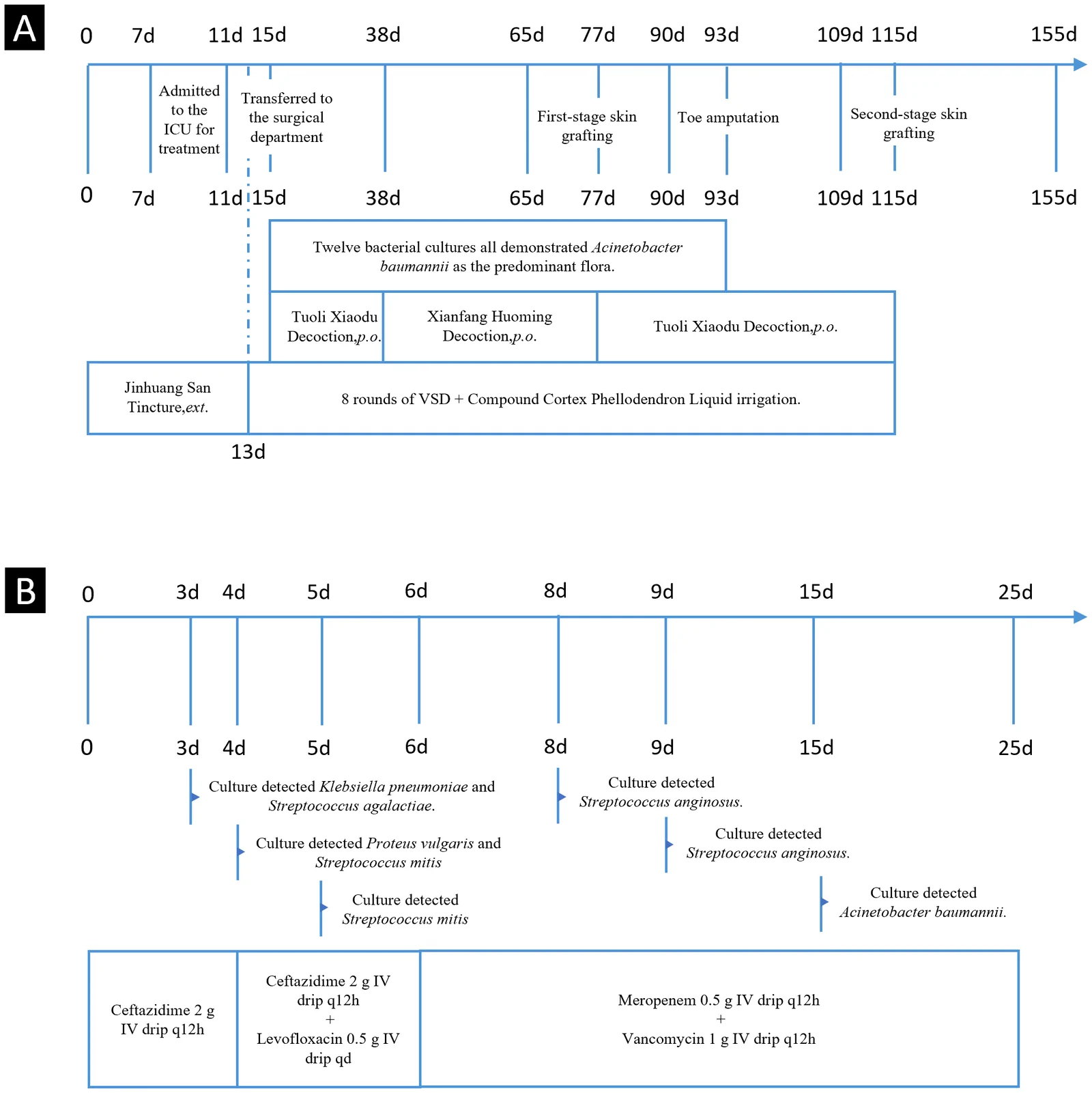
**(A)** Treatment flowchart. **(B)** Timeline of microbiological culture results and antibiotic use during the first 25 days of hospitalization.

From days 13 to 15, vacuum sealing drainage (VSD) was performed in combination with continuous Compound Cortex Phellodendron Liquid irrigation (**Figure 2**). During the same period, *Acinetobacter baumannii* was isolated for the first time from a culture of deep wound exudate. Antimicrobial susceptibility testing showed that the isolate was resistant to most conventional first-line antimicrobial agents (**Table 1**), confirming its classification as extensively drug-resistant *A. baumannii* (XDR-AB). Given the limited availability of effective antimicrobial agents, modified Tuoli Xiaodu Decoction was added orally while standardized wound management was continued.

**Table 1:** Extensively drug-resistant *Acinetobacter baumannii* antimicrobial susceptibility results.

Antibiotic name	MIC (μg/mL)	Susceptibility
Ticarcillin/Clavulanic Acid	≥128	Resistant (R)
Piperacillin/Tazobactam	≥128	Resistant (R)
Ceftazidime	≥64	Resistant (R)
Cefoperazone/Sulbactam	≥64	Resistant (R)
Cefepime	16	Intermediate (I)
Aztreonam	≥64	Resistant (R)
Imipenem	≥16	Resistant (R)
Meropenem	≥16	Resistant (R)
Amikacin	≥64	Resistant (R)
Tobramycin	≥16	Resistant (R)
Ciprofloxacin	≥4	Resistant (R)
Levofloxacin	≥8	Resistant (R)
Doxycycline	≥16	Resistant (R)
Minocycline	8	Intermediate (I)
Tigecycline	4	Intermediate (I)
Colistin	≤0.5	Intermediate (I)
Trimethoprim-Sulfamethoxazole	≥320	Resistant (R)

a Antimicrobial susceptibility interpretations were based primarily on the Clinical and Laboratory Standards Institute (CLSI) M100 guidelines (31st ed., 2021). b CLSI M100 does not provide interpretive breakpoints for tigecycline against Acinetobacter spp. The isolate had a minimum inhibitory concentration (MIC) of 4 μg/mL and was reported as intermediate (I) by the clinical laboratory. c According to CLSI M100, no susceptible (S) category is defined for polymyxins against Acinetobacter spp.; isolates with MICs ≤2 μg/mL are categorized as intermediate (I), whereas those with MICs ≥4 μg/mL are categorized as resistant (R). Accordingly, the isolate, with an MIC ≤0.5 μg/mL, was categorized as intermediate (I).

**Figure 3:**
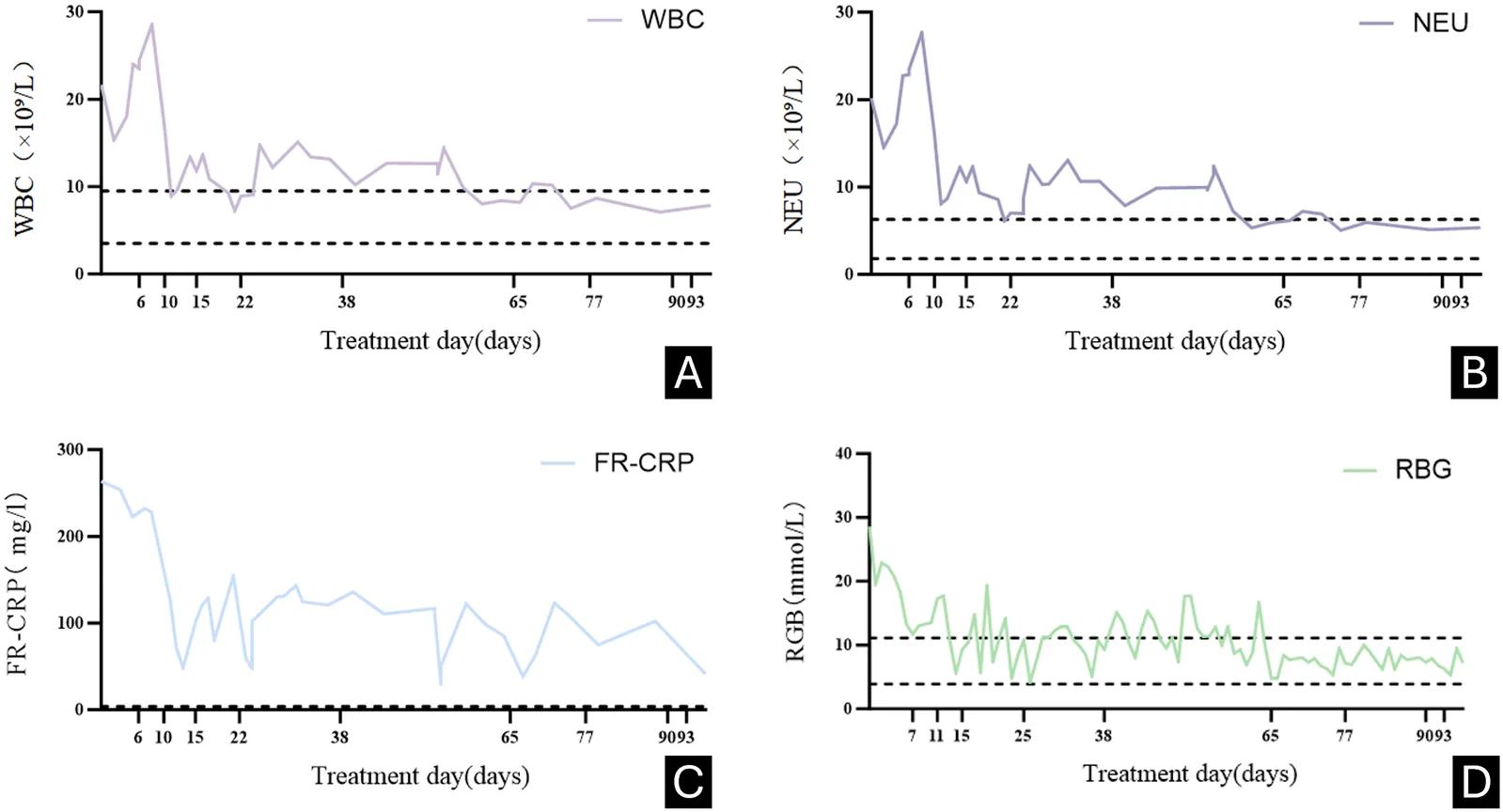
**(A)** Trend of white blood cell count. (reference range: 3.5-9.5 ×10^9^/L). **(B)** Trend of neutrophil count. (reference range: 1.8-6.3 ×10^9^/L). **(C)** Trend of C-reactive protein. (normal limit: < 4 mg/L). **(D)** Trend of random blood glucose (target range for patients with diabetes: 3.9–11.1 mmol/L).

By day 25, three consecutive wound cultures had yielded XDR-AB; however, the patient’s systemic infection had been controlled and the local wound condition was stable. Based on the IWGDF/IDSA guideline (12) for diabetic foot infections and the patient’s clinical condition, antibiotic therapy was discontinued. A coordinated strategy combining Western medicine and traditional Chinese medicine was continued, including oral herbal medicine and, according to the wound condition, repeated VSD combined with Compound Cortex Phellodendron Liquid irrigation and drainage, as well as stepwise debridement. Thereafter, although XDR-AB continued to be isolated from five consecutive wound cultures, the patient’s white blood cell count level returned to their normal ranges (**Figure 3**), and healthy granulation tissue gradually formed in the wound (**Figure 1D**). On day 38, the herbal prescription was adjusted to modified Xianfang Huoming Decoction.

As the infection was effectively controlled and the wound condition improved, regional skin grafting was performed on day 65 (**Figures 1E–G**), and all grafts survived. On day 73, wound culture from the nongrafted area remained positive for *A. baumannii*; however, no local erythema, swelling, malodor, or abnormal exudate was observed. On day 77, renewed concern arose for residual or recurrent osteomyelitis of the affected phalanges, although the systemic infection remained clinically controlled. The oral herbal prescription was adjusted to modified Tuoli Xiaodu Decoction. Osteotomy of the affected phalanges and repeat debridement were performed on day 90, followed by continued VSD combined with Compound Cortex Phellodendron Liquid irrigation. Second-stage skin grafting was performed on day 109 (**Figures 1H, I**). By day 115, both grafts had completely survived, the wound had healed well, and the systemic infection markers had returned to normal. The patient was subsequently discharged (**Figure 1J**).

After discharge, outpatient follow-up and treatment were continued for 40 days, and complete epithelialization of the right lower-extremity wound was achieved on day 155 (**Figures 1K, L**). At the 3-month postoperative follow-up, no wound recurrence or new ulceration was observed. The structure and basic weight-bearing function of the affected limb were preserved, and the patient regained the ability to walk independently on level ground.

During treatment and follow-up, the patient underwent regular monitoring of complete blood counts, liver and renal function, and wound status (**Table 2**). No apparent adverse reactions related to antimicrobial therapy, VSD, or traditional Chinese medicine interventions were observed, and no treatment modification, interruption, or discontinuation occurred because of treatment-related safety concerns.

**Table 2:** Serial changes in liver and renal function during hospitalization.

Hospital day	ALT	AST	ALP	TBIL	ALB	BUN	SCr	eGFR*
	*U/L*	*U/L*	*U/L*	*µmol/L*	*g/L*	*mmol/L*	*µmol/L*	*mL/min/1.73 m²*
1	14	17	101	7.12	28.60	8.09	74	100.6
6	-	-	-	-	-	19.46	107	68.9
11	-	-	-	-	-	15.97	59	107.7
13	34	26	128	20.07	28.00	12.69	51	112.5
21	22	18	122	16.80	35.10	-	-	-
26	76	59	145	9.06	33.00	4.86	42	119.3
29	33	15	155	13.27	30.80	-	-	-
52	22	15	144	6.87	27.80	3.26	46	116.1
56	27	20	149	5.72	30.60	-	-	-
67	25	20	124	4.12	27.10	6.87	50	113.2
89-90	8	9	84	6.56	31.40	7.19	48	114.6

ALP, alkaline phosphatase; ALT, alanine aminotransferase; AST, aspartate aminotransferase; TBIL, total bilirubin; ALB, albumin; BUN, blood urea nitrogen; SCr, serum creatinine; eGFR, estimated glomerular filtration rate; VSD, vacuum sealing drainage; TCM, traditional Chinese medicine.

This case report was prepared in adherence to the CARE guidelines.

## 3 Discussion

This case illustrates the complex clinical challenges posed by the coexistence of a diabetic foot ulcer (DFU), necrotizing fasciitis (NF), and persistent recovery of extensively drug-resistant *Acinetobacter baumannii* (XDR-AB) from wound cultures. The rapid extension of deep infection, increased risk of sepsis, and limited antimicrobial options place such patients at high risk of major amputation (13, 14). Nevertheless, through surgical source control, intensive care support, staged wound management, and comprehensive adjunctive interventions, wound closure was ultimately achieved, with restoration of basic weight-bearing function and independent ambulation. The primary value of this case is not to demonstrate the efficacy of any single intervention, but to present the entire process of acute infection control, adjustment of antimicrobial strategies, and wound reconstruction based on dynamic infection assessment in the setting of persistent XDR-AB recovery.

Early recognition and adequate source control are critical for improving outcomes during the acute phase of NF. The early clinical manifestations of NF are often nonspecific, and local skin changes may underestimate the extent of deep fascial involvement. Although wet gangrene and soft-tissue gas were already present on admission in this case, the infection initially appeared to be largely confined to the foot. As the local swelling extended proximally, inflammatory markers remained abnormal, and circulatory failure developed, the team expanded the scope of surgical exploration, debridement, and drainage, ultimately confirming NF involving the lower extremity. This course indicates that, in patients with diabetic foot infections, when local findings are inconsistent with the systemic response or the condition progresses rapidly within a short period, the extent of infection should be reassessed promptly, and adequate surgical source control should be implemented as early as possible (15). Relatively conservative local management was initially adopted in this case because the patient had anemia, hypoalbuminemia, and a poor overall condition at admission, and extensive debridement could have increased the perioperative risk. However, the subsequent progression of infection suggests that, in patients with suspected NF, even when surgical risks are present, the extent of infection should be defined promptly and the source-control strategy optimized on the basis of a comprehensive assessment.

After NF had been brought under interim control, the persistent recovery of XDR-AB created a new therapeutic decision-making challenge. Colonization with multidrug-resistant organisms is common in open wounds, and culture positivity alone cannot distinguish persistent colonization from active infection (16, 17). Microbiological findings must therefore be interpreted in conjunction with local signs of infection, the systemic inflammatory response, changes in laboratory parameters, and the effectiveness of surgical source control. In this case, the persistent recovery of XDR-AB may have reflected wound colonization, although low-level persistent infection could not be completely excluded. Because quantitative tissue cultures and molecular strain typing were unavailable, these possibilities could not be further distinguished. Nevertheless, during subsequent treatment, local erythema, malodor, and exudation gradually improved, the white blood cell count and C-reactive protein level returned to normal, and skin grafting was ultimately completed. These findings suggest that a positive wound culture should not serve as the sole basis for continuing systemic antimicrobial therapy.

Antimicrobial susceptibility testing showed that the isolate was resistant to most conventional antimicrobial agents, with only limited *in vitro* activity observed for minocycline, tigecycline, and polymyxins. However, the use of these agents in this case remained uncertain. Although minocycline has activity against A. baumannii, evidence supporting its use as monotherapy for invasive infection at a high minimum inhibitory concentration is limited. Established susceptibility breakpoints for tigecycline against A. baumannii are lacking, and its pharmacodynamic target attainment and clinical benefit remain uncertain. Although polymyxins demonstrated *in vitro* activity, they have a narrow therapeutic window and carry a risk of nephrotoxicity. After considering the patient’s infection status, the effectiveness of source control, antimicrobial susceptibility results, and potential treatment risks, the team discontinued systemic antimicrobial therapy. It should be emphasized that this decision does not demonstrate that XDR-AB requires no treatment. Rather, it indicates that after adequate source control and clinical improvement of the infection, persistent culture positivity does not necessarily represent ongoing active infection or mandate prolongation of systemic antimicrobial therapy.

Once infection control had stabilized, the focus of treatment shifted to wound-bed preparation and functional reconstruction. Serial debridement, VSD, wound management, glycemic control, and nutritional support collectively improved the wound condition. Previous studies have suggested that integrated traditional Chinese and Western medicine strategies may have clinical value in the management of DFUs and complex wounds (18, 19). In this case, traditional Chinese medicine interventions were incorporated as part of the comprehensive treatment strategy. Their potential effects may have been related to modulation of the inflammatory response and improvement of the local tissue-repair environment. However, because debridement, VSD, metabolic control, and other treatments were administered concurrently, the independent contribution of traditional Chinese medicine to the final outcome cannot be determined. As the infection-related indicators improved, the wound bed matured, and vascular ultrasonography demonstrated adequate local perfusion (arterial flow velocities: 108, 89, 82, and 78 cm/s; anterior tibial artery not visualized) for reconstruction, staged skin grafting was performed. Complete graft take was achieved after both procedures, ultimately resulting in wound closure and functional limb salvage. This outcome suggests that, on the basis of adequate infection control, wound-bed preparation, and perfusion assessment, persistent recovery of a drug-resistant organism does not necessarily constitute an absolute contraindication to wound reconstruction.

This case has several limitations. First, as a single-case report, multiple interventions—including debridement, VSD, metabolic control, antimicrobial adjustment, and adjunctive traditional Chinese medicine treatment—overlapped temporally, making it impossible to distinguish their individual contributions. Second, because quantitative tissue cultures and strain typing were unavailable, it was not possible to determine whether the persistent recovery of XDR-AB represented colonization, low-level infection, or repeated colonization. In addition, the findings from a single case cannot be directly extrapolated to other patients with XDR-AB diabetic foot infections. In conclusion, this case provides a clinical observation that, on the basis of adequate source control, dynamic infection assessment, and standardized wound management, functional limb salvage may still be achievable in patients with complex diabetic foot infections despite persistent recovery of a drug-resistant organism from the wound. Future multicenter studies are needed to further validate this comprehensive management strategy and clarify the potential role of traditional Chinese medicine interventions within it.

## Data Availability

The original contributions presented in the study are included in the article/supplementary material. Further inquiries can be directed to the corresponding authors.
